# DNA damage-induced ubiquitylation of proteasome controls its proteolytic activity

**DOI:** 10.18632/oncotarget.1060

**Published:** 2013-06-29

**Authors:** Tatiana N. Moiseeva, Andrew Bottrill, Gerry Melino, Nickolai A. Barlev

**Affiliations:** ^1^ Institute of Cytology, Russian Academy of Sciences, St-Petersburg, Russia; ^2^ Molecular Pharmacology Laboratory, Techological University, St-Petersburg, Russia; ^3^ Department of Radiation Oncology, University of Pittsburgh School of Medicine, Hillman Cancer Center, Pittsburgh, PA; ^4^ Department of Biochemistry, University of Leicester, Lancaster Road, Leicester, UK; ^5^ MRC Toxicology Unit, Leicester University, Lancaster Road, Leicester, UK

**Keywords:** proteasome, DNA damage, proteolytic activities, doxorubicin, ubiquitylation

## Abstract

Stability of proteins is largely controlled by post-translational covalent modifications. Among those, ubiquitylation plays a central role as it marks the proteins for proteasome-dependent degradation. Proteolytic activities of proteasomes are critical for execution of various cellular processes, including DNA damage signaling and repair. However, very little is known about the regulation of proteasomal activity in cells during genotoxic stress. Here we investigated post-translational modifications of the 20S proteasomal subunits upon DNA damage induced by doxorubicin. Using mass-spectrometry, we found novel sites of phosphorylation and ubiquitylation in multiple proteasome subunits upon doxorubicin treatment. Ectopic co-expression of proteasome subunits and tagged ubiquitin confirmed the presence of ubiquitylated forms of PSMA5, PSMA1, PSMA3 and PSMB5 in cells. Moreover, we demonstrated that ubiquitylation *in vitro* inhibited chymotrypsin-like and caspase-like activities of proteasomes. *In vivo*, doxorubicin increased the activity of proteasomes, paralleling with attenuation of the overall level of proteasome ubiquitylation. Collectively, our results suggest a novel mechanism whereby the proteolytic activities of proteasomes are dynamically regulated by ubiquitylation upon DNA damage.

## INTRODUCTION

Proteasomes are multi-protein complexes, widely known to participate in protein degradation by ubiquitin-dependent and ubiquitin-independent proteolysis. The 26S proteasome consists of the 20S proteolytic particle and the 19S regulator. The 20S proteasome is a barrel-shaped structure formed of four heptameric rings: the two outer rings are made of alpha-type subunits and the two inner rings are made of beta-type subunits. Beta-subunits are responsible for proteolysis, while the main function of alpha-rings is to regulate an access of a substrate to the proteolytic chamber [1,2].

Since efficient protein degradation is critical for cell cycle progression and apoptosis [3], proteasome inhibitors are considered to be potent anti-cancer drugs and several of them are currently in clinical trials [4]. Bortezomib (PS431) was the first proteasome inhibitor to be approved by FDA for the treatment of multiple myeloma [5]. Recently, a second proteasome inhibitor, arfilzomib, was approved for treatment of the same disease [6]. This preliminary success makes proteasomes an appealing potential therapeutic target and highlights the importance of studying the regulatory mechanisms that control proteasome activities. Moreover, inhibition of interactions between specific ubiquitin ligases and their protein targets ultimately results in abrogation of their ubiquitin-dependent proteasomal degradation, and hence is also considered a promising therapeutic strategy [7, 8, 9].

A number of studies aimed to characterize the role of posttranslational modifications in regulation of proteasome activities. For example, changes in the pattern of covalent modifications was observed in proteasomes from various disease cells, including neurodegenerative diseases [10, 11], coronary occlusion/reperfusion [12] and age-related macular degeneration [13,14]. Collectively, these data suggest that aberrant physiological pathways can alter the activity of proteasomes via post-translational modifications. Phosphorylation of alpha-type subunits in the 20S core particle was shown to regulate proteolytic activities and intercellular localization of proteasomes [reviewed in 15]. Accordingly, all seven alpha-type subunits were found phosphorylated at least on one site in human cells under various conditions [16, 17, 18, 19, 20, 21, 22]. In most of the cases serine phosphorylation was the most abundant modification, but phosphorylation of threonine and tyrosine residues was also reported [15]. Another common modification of proteasome subunits is acetylation. The N-terminal and lysine acetylation were detected in many 20S and 19S proteasome subunits [16, 23]. It was also speculated that acetylation may play an important role in opening the gate of the 20S proteasome chamber thus regulating substrate access [24, 25]. Ubiquitylation is the least studied among known proteasome covalent modifications. This may be due to technical difficulties in registering the ubiquitylated subunits as they may undergo rapid turnover mediated by deubiquitylating enzymes associated with the proteasome (for review see [26]). Despite several proteome-wide screens that showed ubiquitylation of all the 20S proteasome subunits, the biological role of this modification remains unclear.

Doxorubicin (Dox) is an anti-cancer drug that belongs to the family of anthracycline inhibitors of topoisomerase II [27]. Dox was reported to regulate the ubiquitin-proteasome system [28] and enhance the degradation of some transcription factors [29, 30]. Moreover, proteasomes were shown to interact with Dox directly and carry it from cytoplasm into the nucleus [31].

In this study we show that cellular DNA damage induced by doxorubicin leads to changes in phosphorylation and ubiquitylation states of several proteasomal subunits and affects the proteolytic activities of proteasomes both *in vitro* and *in vivo*.

## RESULTS

### DNA damage elicits post-translational modifications on several 20S proteasome subunits

**Figure 1:**
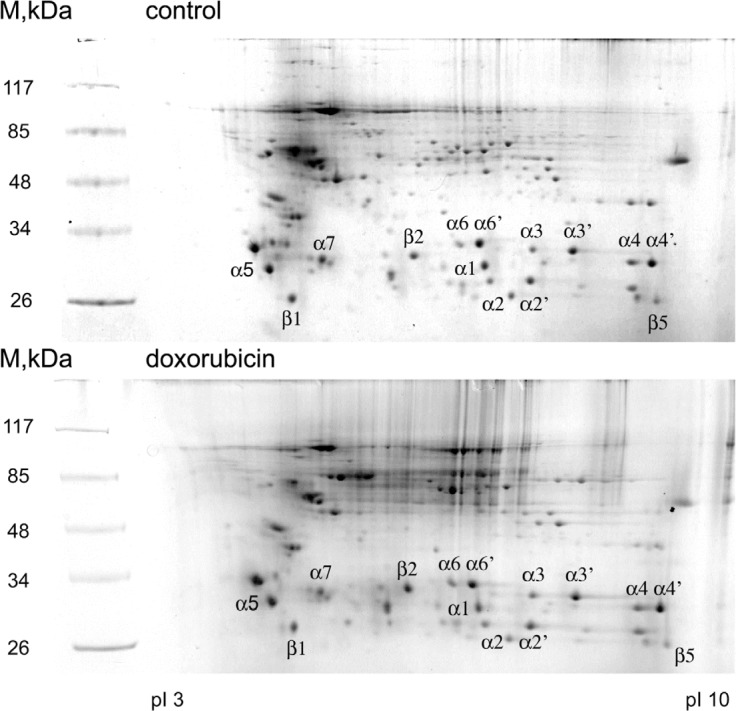
2D electrophoregrams of proteasomal proteins before and after doxorubicin treatment Proteasomes were extracted from control (A) and doxorubicin treated (B) K562 cells and then separated on 2D-gel (isoelectric focusing followed by SDS-PAGE). Separated proteins were visualized by Coomassie staining. Marked spots were excised from the gel and analyzed by mass-spectrometry.

Several studies suggest that proteasomes play an important role in DNA damage signaling and DNA repair [32]. An important question emerges as whether the activity of proteasomes is regulated upon DNA damage and what is the molecular mechanism behind it. To address this, we first decided to catalogue the posttranslational modifications of 20S proteasome subunits induced by doxorubicin-mediated genotoxic stress by using a proteomic approach. To this end, proteasomal proteins purified from control and DNA damage treated cells were separated by 2D-electrophoresis (Fig. 1) and spots, corresponding to the 20S subunits were excised from the gel and analyzed using LC-MS/MS mass-spectrometry. As shown in Table 1, we were able to identify both novel and previously reported sites for phosphorylation, acetylation and ubiquitylation. The majority of newly identified modification sites are acetylated amino acids including lysines, serines and alanines. In addition, we identified phosphorylation sites on all of the alpha-type subunits, except PSMA7. Finally, two new ubiquitylation sites – Lys15 on PSMB3 and Lys52 on PSMA3 were also detected. Interestingly, several of the newly found acetylation sites were previously reported to be ubiquitylated (Lys45 on PSMA6, Lys165 on PSMA2 and many more [33]).

**Table 1:** Post-translational modifications of the proteasomal subunits upon DNA damage induced by doxorubicin

Subunit	Control	doxorubicin
PSMA6 (α1)	*Ub: Lys171*	
	Ac: **Ser2, Lys45**, Lys104	Ac: **Ser2, Lys30, Lys45, Lys54**
PSMA2 (α2)	*Ub: Lys53, Lys64*	*Ub: Lys53*
	Ac: **Ala2, Lys165**	Ac: **Ala2, Lys165**
PSMA4 (α3)	*Ub: Lys195, Lys199*	
	Ac: Lys127, Lys195	Ac: Lys187, Lys195, Lys199
PSMA5 (α5)	*Ub: Lys192, Lys196*	*Ub: Lys196, Lys203*
	P: Ser16	P: Ser16, Thr55, Ser56, **Thr213 or Thr219**
PSMA1 (α6)	*Ub: Lys115*	*Ub: Lys30, Lys115, Lys189, Lys208*
	Ac: **Met1, Lys30, Lys39, Lys61**	Ac: **Met1, Lys50, Lys115**
		P: **Tyr6, Thr11**
PSMA3 (α7)	*Ub: Lys52*	
	P: Ser250	P: Ser250; **one of the four Serines (2-8)**
PSMB6 (β1)	Ac: **Lys67**	
	*Ub: Lys67*	
	P: **Thr56, Ser58**, Thr59	P: **Thr56, Ser58**, Thr59
PSMB7(β2)	Ac: **Lys72**, Lys249	
	*Ub: Lys249*	*Ub: Lys249*
PSMB3 (β3)	Ac: **Ser2**, Lys77	Ac: **Ser2, Lys17**
	*Ub: Lys15, Lys17, Lys77*	
PSMB5 (β5)	*Ub: Lys91*	
PSMB1 (β6)		*Ub: Lys94*
PSMB4 (β7)		Ac: **Lys201**
	*Ub: Lys201*	

Proteasomal subunits from normal (control) and DNA damaged cells (doxorubicin) were separated by 2D electrophoresis and analysed by mass-spectrometry. The following modifications are shown: P – phosphorylation, Ub – ubiquitylation (shown in italic), Ac – acetylation. Novel unpublished modification sites are shown in bold.

Doxorubicin treatment caused a significant change in the pattern of modifications of proteasomal subunits compared to the ones purified from non-treated cells. Our data showed that PSMA5, PSMA1, PSMA3 and PSMB6 subunits underwent phosphorylation on multiple sites (Table 1). However, in addition to constitutive phosphorylation of Ser16 in PSMA1, Ser250 in PSMA3, and Thr56, Ser58, and Thr59 in PSMB7, we identified several doxorubicin-induced phosphorylation sites, namely Thr55, Ser56, and Thr213/219 in PSMA5, and Tyr6 and Thr11 in PSMA1 subunits, respectively.

The dynamics of specific covalent modifications on proteasomal proteins was different. While doxorubicin treatment resulted in depositing only the new phosphorylation sites to the target subunits without affecting the constitutively phosphorylated ones, the patterns of doxorubicin-induced ubiquitylation and acetylation were more complex. We found that acetylation of the 20S proteasomal subunits was dynamic so that DNA damage not only induced acetylation of additional lysines (Lys30 and Lys54 of PSMA6, Lys 187 and Lys 199 of PSMA4, Lys 203 of PSMA5 etc.), but also erased the ones acetylated under normal conditions (Lys104 of PSMA6, Lys127 of PSMA4, Lys192 of PSMA5 etc.). Moreover, upon doxorubicin treatment several lysine residues were found to switch their states from acetylated to the ubiquitylated ones (K30 of PSMA1) and vice versa (K199 of PSMA4, K17 of PSMB3, K201 of PSMB4). Also, a mixture of acetylated and ubiquitylated forms of a particular peptide was found in the same sample (K195 of PSMA4, K115 of PSMA1, K67 of PSMB6, K249 of PSMB7, K77 of PSMB3). Overall, the level of ubiquitylation of the 20S subunits, except PSMA1 and PSMB1, decreased upon DNA damage (Table 1).

**Figure 2:**
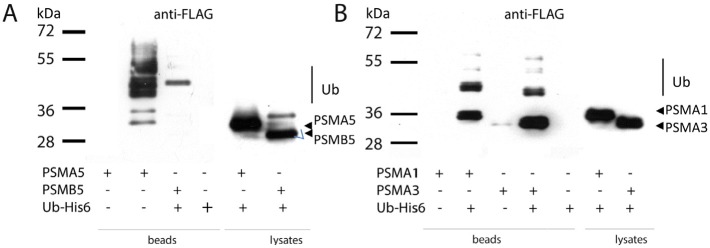
The alpha type subunits of the 20S proteasome undergo ubiquitylation *in vivo* H1299 cells transiently co-expressing one of the indicated FLAG-tagged proteasome subunits and His-tagged ubiquitin were lysed in denaturing conditions. Ubiquitylated proteins were precipitated with Ni-NTA beads, and the ubiquitylated isoforms of PSMA5-FLAG, PSMB5-FLAG (A) and PSMA1-FLAG, PSMA3-FLAG (B) subunits were separated by SDS-PAGE followed by western-blotting with anti-FLAG antibodies. Positions of ubiquitylated subunits are indicated.

To validate the results of mass-spectrometry on ubiquitylated proteasome subunits, we individually examined the ubiquitylation status of ectopically expressed PSMA5, PSMA1, PSMA3 and PSMB5 subunits in the presence of tagged ubiquitin. To this end, whole-cell extracts were prepared from H1299 cells co-transfected with respective FLAG-tagged proteasome subunits and a vector, expressing His-tagged ubiquitin. 6His-ubiquitylated proteins were then affinity purified on the Ni-NTA resin followed by the western blot analysis using FLAG antibodies to detect tagged proteasomal subunits. The results of experiments shown in Fig. 2 suggest that all four subunits investigated were present in various ubiquitylated forms. The PSMB5 subunit was represented mostly as a mono-ubiquitylated species and this modification largely affected the proteolytically non-processed form of PSMB5 (Fig. 2A, compare lanes 4 and 7). The PSMA5 ubiquitylation pattern was more heterogeneous, presumably due to additional non-ubiquitin covalent modifications, such as acetylation, phosphorylation and/or O-glycosylation. These modifications are known to affect the protein mobility in SDS-PAGE. However, the majority of PSMA5 proteins in the presence of ubiquitin was distributed between two zones, which likely correspond to mono- and di-ubiquitylated forms (Fig. 2a, compare lanes 3 and 6). It should also be noted that most of the PSMA5 species retained on Ni^2+^ beads were ubiquitylated, because no PSMA5 protein was detected on beads in the absence of 6His-ubiquitin (Fig. 2A, compare lanes 2 and 3). In the same experiment, PSMA1 and PSMA3 subunits were preferentially mono-ubiquitylated, although di- and tri-ubiquitylated isoforms were also detected but at a lesser extent (Fig. 2B).

### Ectopically expressed proteasome subunits are ubiquitylated

**Figure 3:**
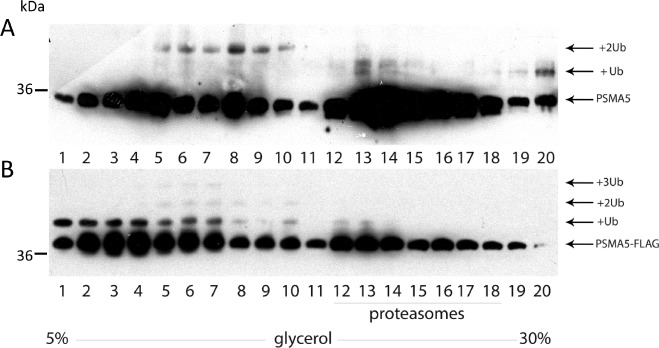
The ubiquitylated PSMA5 subunit is distributed among the protein fractions of different molecular weight Whole-cell extracts prepared from H1299 cells expressing endogenous PSMA5 (A) or ectopic PSMA5-FLAG (B) proteins, were subjected to fractionation in the 5-30% glycerol concentration gradient. 20 equal fractions of different densities were collected and analyzed by western-blotting using anti-PSMA5 (endogenous protein) (A) or anti-FLAG (B) (ectopic PSMA5 protein) antibodies.

Next, we decided to compare the ubiquitylation patterns of the monomeric PSMA5 subunit versus the one incorporated into the 20S core particle. Thus, H1299 cell extracts containing either endogenous PSMA5 or ectopic PSMA5-FLAG proteins in various proteasomal complexes were separated in glycerol density gradients according to their molecular weights followed by western-blotting using anti-PSMA5 (Fig. 3A) or anti-FLAG antibodies (Fig. 3B), respectively. Both ectopic and endogenous PSMA5 proteins were found in multiple gradient fractions. Specifically, the endogenous PSMA5 protein was observed both in the low molecular weight (LMW) fractions and in the high molecular weight (HMW) fractions. Apparently, the PSMA5 species that belong to the LMW fractions represent a mix of free monomeric subunits, its dimers and possibly a complex with PSMA1 and PSMA3 subunits. The PSMA5 species found in the HMW fraction likely correspond to the full-sized 20S proteasome and its interacting proteins (Fig. 3, A and B). Importantly, endogenous PSMA5 was preferentially di-ubiquitylated in the LMW fraction, whereas PSMA5 in the 20S proteasome HMW fraction was mostly mono-ubiquitylated (Fig. 3A). A similar pattern was observed for the ectopic PSMA5-FLAG protein with the exception that in the LMW fractions it was not only di-ubiquitylated, but also mono- and tri-ubiquitylated (Fig. 3B). Collectively, our results suggest that both endogenous and ectopic alpha-type subunits undergo ubiquitylation in cells. Moreover, the neighbouring subunits of the 20S particle likely restrict the extent of ubiquitylation for PSMA5 to only mono-ubiquitylation. On the contrary, PSMA5 in its free, or heterotrimeric form, is more susceptible to ubiquitylation.

### Ubiquitylation in vitro inhibits peptidase activities of the proteasome

**Figure 4:**
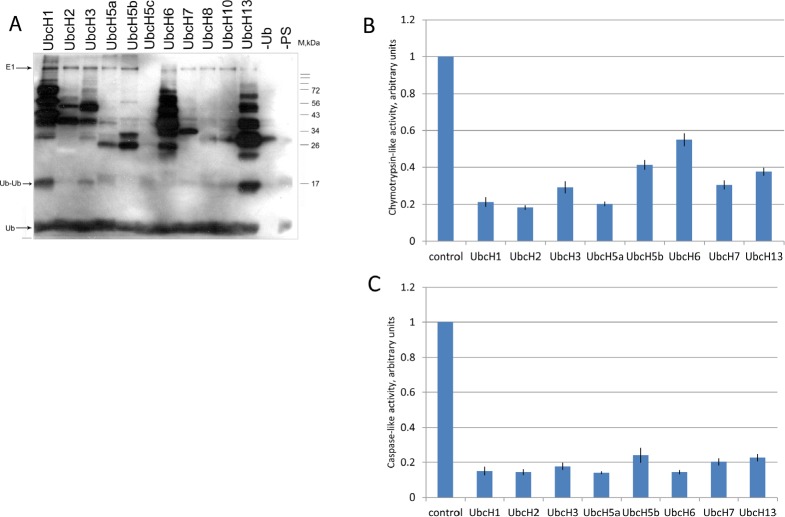
Ubiquitylation of proteasomes *in vitro* inhibits their proteolytic activities **A.** Proteasomes extracted from untreated K562 cells were ubiquitylated in vitro using biotinylated ubiquitin and different E2 enzymes: UbcH1, UbcH2, UbcH3, UbcH5a, UbcH5b, UbcH5c, UbcH6, UbcH7, UbcH8, UbcH10, and UbcH13. Samples were then subjected to SDS-PAGE followed by transfer to nitrocellulose membrane. Ubiquitylated proteins were detected using the streptavidin-biotin system. The two right lanes represent control reactions without ubiquitin or without proteasomes. **B and C.** Proteasomes were ubiquitylated as described above in the presence of either UbcH1, UbcH2, UbcH3, UbcH5a, UbcH5b, UbcH6, UbcH7 or UbcH13 ubiquitin-conjugating enzymes followed by the measurement of chymotrypsin-like (B) and caspase-like (C) activities using fluorogenic peptides as specific substrates. In each case, activity of the non-ubiquitylated proteasome was arbitrary set as 1. Peptidase activities of each sample were tested in triplicates. Standard deviations are shown.

To assess the effect of ubiquitylation on the proteolytic activities of the 20S complex, we performed *in vitro* ubiquitylation assay followed by the peptidase activity assay (Fig 4). A collection of purified E2 ubiquitin conjugating enzymes (Enzo Life Sciences) was tested for their ability to ubiquitylate 20S proteasomes isolated from K562 cells (Fig. 4A). From eleven E2 ligases tested eight (UbcH1, UbcH2, UbcH3, UbcH5a, UbcH5b, UbcH6, UbcH7, and UbcH13) exhibited ubiquitylation activity towards the 20S proteasomal proteins (Fig. 4A). Those eight ligases were subsequently used for ubiquitylation of the 20S complex followed by the measurement of caspase-like and chymotrypsin-like activities of proteasomes (Fig. 4B, C). The results of experiment showed that ubiquitylation by all E2 enzymes inhibited to a various extent both caspase-like and chymotrypsin-like activities of proteasomes (Fig. 4B and 4C, respectively). Interestingly, there was no strict correlation between the number of ubiquitylated subunits and the efficiency of repression. This suggests that mono-ubiquitylation of even one subunit of the 20S complex may result in strong inhibitory effect.

### DNA damage augments the proteolytic activities of proteasomes in vivo

**Figure 5:**
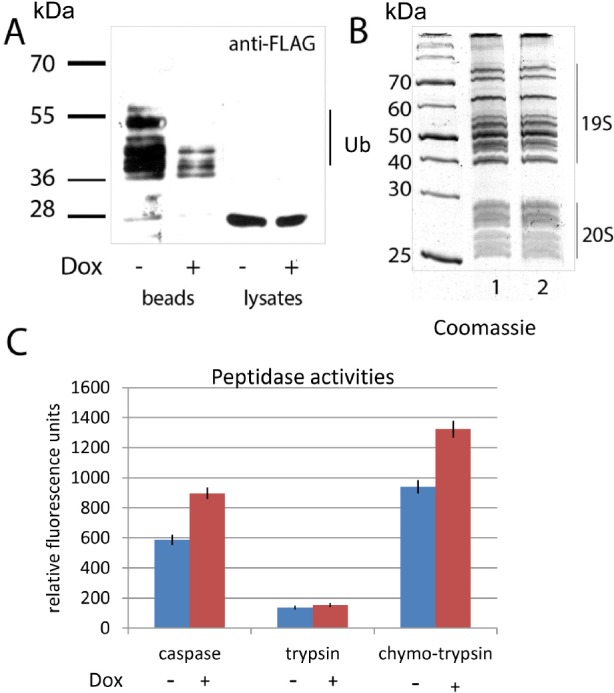
Doxorubicin treatment enhances the activity of proteasomes concomitant with a decrease in ubiquitylation **A.** H1299 cells, ectopically expressing PSMA5-FLAG and His-tagged ubiquitin were treated with doxorubicin or DMSO for 6 hours. Cells were lysed in denaturing conditions. Ubiquitylated proteins were precipitated with Ni-NTA beads, and the ubiquitylated isoforms of PSMA5-FLAG were detected by western blotting with M2 anti-FLAG antibodies. Positions of ubiquitylated isoforms of the PSMA5-FLAG protein are shown. **B.** 26S proteasomes were purified from K562 cells non-treated (lane 1) or treated with doxorubicin (lane 2). Resulting proteins were separated by SDS-PAGE and visualized by Coomassie staining. Positions of 19S and 20S sub-complexes in the gel are shown. **C.** Proteasomes extracted from K562 cells non-treated (white bars) or treated with doxorubicin (shaded bars), were compared for their peptidase activities (trypsin-like, chymotrypsin-like and caspase-like) using specific fluorogenic peptides. Peptidase activities of each sample were tested in triplicates. Standard deviations are shown.

Our mass-spec results in K562 cells suggested that DNA damage decreased the overall level of ubiquitylation of the 20S subunits. We wanted to verify the mass-spec data in respect to whether doxorubicin treatment indeed altered the level of proteasome ubiquitylation *in vivo* using the PSMA5 subunit as an example (Fig. 5A). Thus, we compared the levels of ubiquitylation of ectopically expressed PSMA5 in H1299 cells treated or non-treated with doxorubicin. To enrich the population of ubiquitylated proteins including PSMA5, cells were co-transfected with 6His-ubiquitin followed by the chelate chromatography on Ni-NTA beads. As evidenced from Fig. 5A, the overall level of ubiquitylated PSMA5 decreased significantly in cells treated with doxorubicin compared to control cells. Note, that the cellular levels of PSMA5 in both types of cells were comparable (Fig. 5A). These results suggest that ubiquitylation unlikely targets the PSMA5 protein for degradation but rather plays a regulatory role.

We hypothesized that since the proteolytic activities of proteasomes are inhibited by ubiquitylation, and doxorubicin treatment, in turn, decreased the level of ubiquitylation, then doxorubicin should augment the proteolytic activities of proteasomes. To validate this hypothesis, we tested proteasomes, extracted from the control and doxorubicin-induced K562 cells for trypsin-like, chymotrypsin-like and caspase-like activities using fluorogenic peptide substrates (Fig. 5B and 5C). As can be judged from the picture of electrophoresis (Fig. 5B) the amounts of purified proteasomes from treated and non-treated cells were comparable. However, both caspase-like and chymotrypsin-like peptidase activities of proteasomes purified from doxorubicin treated cells were ~30% higher than from control cells (Fig. 5C). Importantly, trypsin-like activity was comparable in both cases pointing to a selectivity of effect mediated by doxorubicin. Collectively, the results obtained support our hypothesis that DNA damage controls the activity of proteasomes by modulating the level of their ubiquitylation.

## DISCUSSION

26S proteasomes play a central role in regulated intracellular protein degradation and therefore represent an appealing therapeutic target. Accordingly, two proteasome inhibitors bortezomib and arfilzomib proved successful in treating several malignancies, including multiple myeloma (MM). However, these drugs were already reported to elicit drug resistance in MM patients [34]. In this respect it should be noted that MM patients refractory to bortezomib monotherapy, benefited from the combination treatment with bortezomib and DNA damaging drug, doxorubicin [35]. Thus, uncovering novel mechanisms of proteasome regulation should facilitate the development of new approaches for the proteasome-targeted cancer therapy.

Here we show that genotoxic stress induced by doxorubicin causes alterations in the pattern of proteasome post-translational modifications augmenting its proteolytic activities.

We established that DNA damage induced phosphorylation of several alpha-type subunits (PSMA5, PSMA1, and PSMA3) of the 20S core proteasome complex. Phosphorylation increases net negative charge of proteasome subunits and hence may affect protein-protein interactions and the proteasome gate opening. In this respect, it has been shown that phosphorylation of the PSMA3 subunit affected the efficiency of ubiquitin-independent degradation of several proteins [36, 37]. It has also been demonstrated that phosphorylation modulated the formation of the 26S proteasome complex and its proteolytic activities [38]. Changes in the phosphorylation state of proteasome subunits were detected during the cell cycle transition [39, 40], development of Alzheimer's disease [10] and apoptosis of Jurkat T cells [41]. These changes in phosphorylation were paralleled by changes in the proteolytic activities of proteasomes. Therefore, it can be concluded that phosphorylation affects peptidase activities of proteasomes.

The mass-spectrometry data revealed that 12 out of 14 proteasome core subunits were ubiquitylated. This covalent modification can be easily detected by mass-spectrometry by two additional glycines left from ubiquitin attached to the target protein after trypsin digest, so-called “ubiquitin signature”. Importantly, trypsin-like proteases also present in cells and can remove ubiquitins from the target proteins during cell extract preparation and protein purification [42]. Because of this, we observed the “ubiquitin signatures” on proteins with mass corresponding to non-ubiquitylated proteins. When cell extracts were prepared under denaturing conditions, ubiquitins attached to the target lysines were preserved, supporting our notion that proteasome-independent proteolysis may cleave off ubiquitins during cell extract preparation. Our results suggest that several lysines can be not only ubiquitylated but also acetylated (Table I) in response to DNA damage. These two modifications differ structurally and thus may affect the proteasome properties with different functional outcome. Given that the same lysine residues undergo either ubiquitylation or acetylation, it is conceivable that acetylation and ubiquitylation compete with each other and genotoxic stress may regulate either activity. To support this hypothesis, it has been shown that several E3 ubiquitin ligases, including Mdm2 and COP1, are inhibited upon DNA damage [43, 44], On the contrary, it is known that acetylation exerted by HATs, e.g. p300/CBP, is enhanced by DNA damage [45].

Alternative scenario would imply that DNA damage facilitates the turnover of ubiquitins on proteasomal subunits by augmenting the activity of ubiquitin-specific hydrolases (USPs). In concord with this hypothesis is the fact that several USPs were shown to be phosphorylated by ATM kinase in response to DNA damage [46]. Collectively, our findings suggest that the dynamic balance between acetylation and ubiquitylation may regulate the biological functions of proteasomes in response to genotoxic stress.

The mass-spectrometry data revealed that for many subunits more than one ubiquitylation site was detected. For example, there are four ubiquitylated lysines in the PSMA1 subunit after the induction of genotoxic stress by doxorubicin (Table 1). However, the western-blotting results suggest that the PSMA1 subunit was mostly mono-ubiquitylated according to the shift in the molecular weight (Fig. 2B and data not shown). One explanation to the apparent discrepancy is that the mass-spectrometry analysis dealt with a heterogeneous population of PSMA1 molecules monoubiquitylated on different lysines. In general, monoubiquitylation of proteins regulates various important biological processes, including membrane transport, gene expression and retrovirus budding [47]. It also affects activities of different enzymes (e.g. DNA polymerase eta [48], phospholipase D1 [49]) and transcriptional regulators [50]. Thus, it is conceivable that monoubiquitylation also regulates the catalytic activities of proteasomes without their degradation.

Peptidase activities of proteasomes are exerted by three beta-type subunits: PSMB6 possesses the caspase-like activity, PSMB7 is responsible for the trypsin-like activity, and PSMB5 mediates the chymotrypsin-like activity. Mono-ubiquitylation may affect the proteolytic activities of proteasomes in several ways: while covalent attachment of ubiquitins to the catalytic beta-type subunits is expected to directly modulate their peptidase activities, ubiquitylation of alpha-type subunits likely affects the activities indirectly, via altering the passage of the target polypeptide into the proteolytic chamber.

A significant portion of the alpha-type subunits exists in a proteasome-free form and apparently exists and exerts different biological functions. Interestingly, we found ubiquitylated isoforms of PSMA5 both incorporated into the proteasome core and in LMW fractions. Noticeable amounts of the free PSMA5 subunit observed in the cell extract might be explained by the fact that PSMA5 plays a key role in triggering assembly of a proteasome alpha-ring [51]. Therefore, ubiquitylation of free PSMA5 could regulate its interactions with other proteins and/or play role in proteasome maturation. Since proteasome complexes possess multiple enzymatic activities including the endoribonuclease one [52, 53, 54], it would be interesting to investigate whether ubiquitylation also affects the ability of proteasomes to participate in metabolism of RNA. Since proteasome-specific E3 ubiquitin ligases are not known yet, it is impossible to accurately assess *in vitro* whether proteasomes are exclusively mono-ubiquitylated or can be also poly-ubiquitylated. However, our *in vitro* experiments with E1 and various E2 enzymes clearly showed that ubiquitylation decreased caspase-like and chymotrypsin-like activities of purified proteasomes (Fig.4 B and C). In accord with this notion, the LC/MS-MS data on proteasomes purified from doxorubicin-treated and untreated cells suggested that the level of proteasome ubiquitylation decreased upon DNA damage. Importantly, this attenuation of ubiquitylation was concomitant with an increase in proteolytic activities. Therefore, we propose a model whereby DNA damage modulates the proteolytic activity of proteasomes by attenuating their ubiquitylation.

## MATERIAL AND METHODS

### Cell cultivation

K562 cells were cultivated in RPMI 1640 media, containing 10% embryonic calf serum. H1299 cells were cultivated in DMEM media, containing 10% embryonic calf serum and penicillin/streptomycin. To induce DNA damage, the cells were incubated with 4μM doxorubicin for 24 hours or with 6μM doxorubicin for 4 hours.

### Preparation of whole-cell extracts

K562 cells were washed with PBS solution and then incubated in PBS, containing 0.1 % NP-40, 5 mM PMSF and 100 μM leupeptine, for 30 minutes at +4°C with shaking. Then the lysate was sonicated 5-6 times for 30 seconds to shear chromatin, following by centrifugation for 15 minutes to remove cell debris. The protein concentration was determined by Bradford assay.

### Extraction of proteasomes

Proteasomes were extracted using a multistep procedure as follows: the cytoplasmic fraction of K562 cells was separated from nuclei by centrifugation in 1.7M sucrose and then precipitated by ammonium sulfate (40% of saturation). Precipitated proteins were resuspended in buffer 1 (20mM Tris-HCl pH 7.5, 5mM MgCl_2_, 1mM ATP, 83mM KCl, 17mM NaCl) and dialyzed against the same buffer. The sample was subjected to ultracentrifugation in sucrose gradient (15%-30%) and the fractions with corresponding density were subsequently separated by DE-52 anion-exchange chromatography. Fractions, containing proteasomes, were collected and precipitated with ethanol. Precipitates were then resuspended in the reaction buffer and the protein concentration was measured with Bradford solution.

### Protein electrophoresis and western-blotting

Isoelectric focusing for 2D-electrophoresis was performed in IPG – dry strips according to the manufacturer's instructions (GE Healthcare). SDS-PAGE was then carried out in 15% PAG and the resolved proteins were stained with Coomassie. To verify the identities of proteasomal proteins by western, the proteins were transferred onto PVDF membrane following overnight incubation with primary anti-PSMA5, anti-PSMA1, anti-PSMA3, anti-PSMB6 or anti-biotin antibodies (EnzoLifesciences, UK).

### Mass-spectrometry for protein identification

The Coomassie-stained spots, containing proteins of interest, were excised from the gel. Each spot was destained and digested with trypsin using an automated digest robot (Multiprobe II Plus EX, Perkin Elmer, UK). Liquid Chromatography (LC) prior to mass-spectrometry (MS) was performed on a reverse-phase trapping column containing Acclaim PepMap media (Dionex, UK) and eluted through a reverse-phase capillary column containing Waters Symmetry C18 100 E media (Waters, UK). The output from the column was sprayed directly into the nanospray ion source of the 4000 Q-Trap mass spectrometer (Applied Biosystems, Warrington, UK). Fragment ion spectra generated by LC-MS/MS were searched using the MASCOT [55] search tool against the UniProtKB/Swiss-Prot [56] protein database using appropriate parameters. Protein identifications were only considered if they contained three or more peptides with p values scores < 0.05.

### Ubiquitylation of proteasomes in vitro

3μg of proteasome preparations form K562 cells or 1 μg of commercial 20S proteasomes (EnzoLifesciences, UK) were ubiquitylated in the presence of biotinylated ubiquitin using the Ubiquitylation kit (EnzoLifesciences, UK) that contained the following E2 enzymes: UbcH1, UbcH2, UbcH3, UbcH5a, UbcH5b, UbcH5c, UbcH6, UbcH7, UbcH8, UbcH10, and UbcH13. The ubiquitylated proteins were detected by western blotting with biotin-specific antibodies.

### Assay on the proteolytic activities of proteasomes

To measure the proteolytic activity of proteasomes the following fluorogenic peptides were used: Benzyloxycarbonyl—L-leucyl-L-leucyl-glutamyl-L-7-amino- 4-methylcoumarin—Bz-L-L-E-AMC (pro-caspase activity) and N-Succinyl-L-leucyl-L-leucyl-L-valyl-L-tyrosine-methylcoumarylamid—Suc-LLVYAMC (chymotrypsin-like) (all purchased from EnzoLifesciences, UK) as described previously [54].

### Identification of overexpressed tagged protein among ubiquitylated proteins

H1299 cells were co-transfected with vectors expressing FLAG-tagged proteasome subunits (PSMA5, mutant 5KR PSMA5, PSMA1, PSMA3 or PSMB5) and His-tagged ubiquitin. 96 hours after transfection cells were lysed in denaturing conditions in buffer B (100 mM NaH_2_PO_4_, 10 mM Tris HCl, 8M urea, pH 8.0), chromatin was sheared by pipetting through needle for 10 times and then precipitated by centrifugation at 13000 rpm for 2 minutes. 30ul of washed Ni-NTA beads (Qiagen) were added to the supernatants. After 2 hours incubation at room temperature with rotation, beads were precipitated, washed 3 times with buffer C (100 mM NaH_2_PO_4_, 10 mM Tris HCl, 8M urea, pH 6.3), and boiled for 7 minutes at 95°C with SDS loading buffer. Ni-bound ubiquitin-containing proteins were separated by SDS-PAGE. Western-blot analysis with anti-FLAG M2 antibody was used to detect overexpressed proteasomal subunits.

### Genetic constructs

PIRES-hrGFP1a vectors, expressing FLAG-tagged proteasome subunits PSMA5, PSMA1, PSMA3 or PSMB5 were kindly provided by Dr. Andrey Nikiforov.
